# Screening Laboratory Requests

**DOI:** 10.3201/eid1211.060711

**Published:** 2006-11

**Authors:** Cathy A. Petti, Christopher R. Polage, David R. Hillyard

**Affiliations:** * University of Utah School of Medicine, Salt Lake City, Utah, USA;; † Associated Regional and University Pathologists Laboratories, Salt Lake City, Utah, USA

**Keywords:** bioterrorism, diagnostic tests, health resources, public health, laboratories, decision making, letter

To the Editor: In August 1999, the Laboratory Response Network (LRN) was established to better integrate and improve laboratory capacity for responding to public health threats (*1*). However, while experts have focused on clinical indications for testing for agents of bioterrorism, laboratory methods for microbial identification, and needs for integrated communication networks (*2**–**4*), little attention has been given to how sentinel laboratories can effectively screen clinicians' requests for testing pathogens designated as global health threats.

In times of crisis, clinicians often pressure laboratorians to perform testing for patients whose probability for disease is very low or for nonvalidated sample types. In 2001, a few cases of anthrax triggered large numbers of nationwide requests to test nasal swabs for Bacillus anthracis despite the absence of data to support this clinical practice outside epidemiologic investigations (*5*). Similarly, a false-positive result for severe acute respiratory syndrome (SARS) in 2003 from the National Microbiology Laboratory in Canada created public alarm that SARS was reemerging, when the virus was actually that of a common respiratory illness in a nursing home (*6*). The problem is further complicated when laboratories other than the LRN lack standardization, have greater access to nucleic acid amplification-based testing, and develop tests for global health threats outside a quality-regulated system. False-positive results caused by contamination or cross-reactivity with a microorganism of low virulence can disrupt a public health system, adversely affect patient care, and increase costs (*6**–**8*); false-negative results may prompt clinicians to discontinue containment procedures and potentially risk transmitting a virulent microorganism. At our sentinel laboratory, we recognized these challenges and took steps to promote judicious use of testing for agents designated as global health threats. We report use of an algorithm to evaluate test requests for SARS-associated coronavirus and highly pathogenic avian influenza H5N1; however, the algorithm can be used to screen testing requests for any pathogen that has potential to threaten public health.

During outbreaks of SARS and H5N1, a laboratory protocol was established to notify the on-call laboratory professional when a sample was received for testing for 1 of these pathogens (Figure). The protocol required the laboratorian to communicate directly with the clinician, using a script with questions based on criteria established by the Centers for Disease Control and Prevention, to determine the medical necessity for testing (*9**,**10*). Samples from patients not meeting these criteria were rejected. Testing for SARS used an in-house real-time PCR assay with a standard laboratory protocol. Samples accepted for H5N1 testing were screened by a nonspecific hemagglutinin influenza PCR assay and, if results were positive, were to be forwarded to an LRN laboratory. Positive results were to be reported only after confirmation by an LRN laboratory. Laboratory professionals were specifically trained about the sensitivity, specificity, positive predictive value, and negative predictive value of test methods in relation to sample type, time between symptom onset and specimen collection, and disease prevalence.

**Figure:**
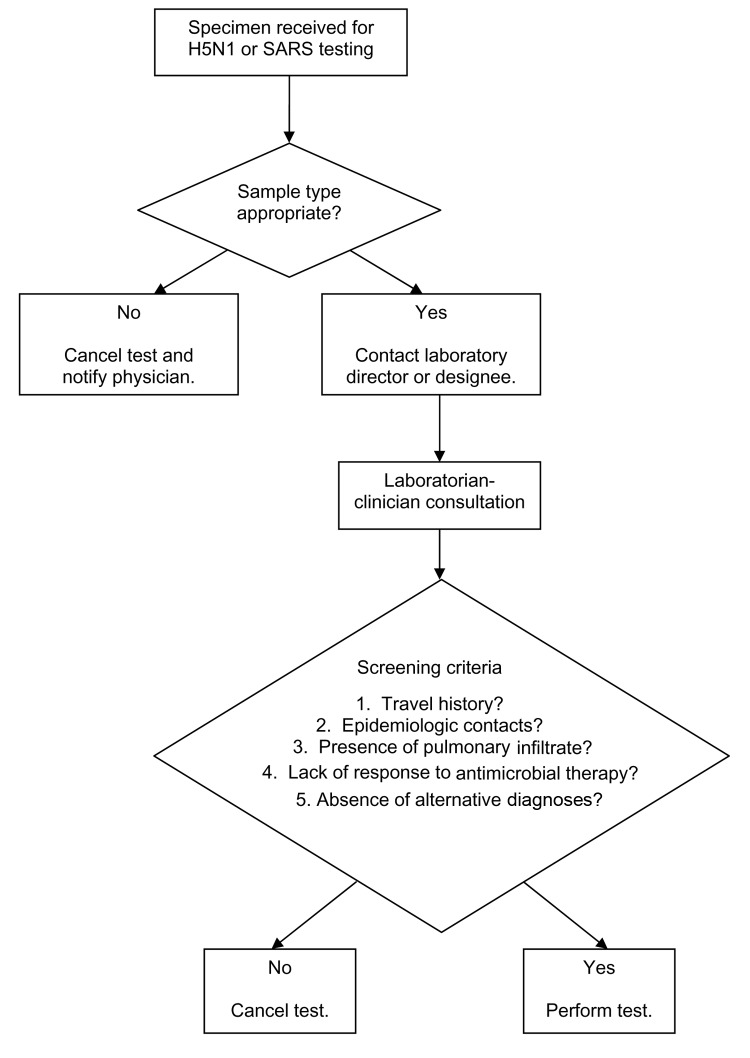
Laboratory algorithm used to screen test requests for avian influenza H5N1 or severe acute respiratory syndrome (SARS).

Of 41 samples (40 SARS and 1 H5N1) received for testing, 26 (63%) samples were not tested because clinician responses failed to satisfy the screening criteria. The remaining 15 (37%) samples met criteria for testing and all had negative results. In the absence of positive results, no confirmatory testing was indicated.

Although SARS no longer poses a credible threat and human-to-human transmission of H5N1 has not been well delineated, our experiences with these 2 pathogens demonstrate how a sentinel laboratory can effectively intervene in the initial phases of a public health threat. We found that having a laboratory professional contact the clinician and systematically ask the scripted questions was a pragmatic tool for the first phase of response and resulted in cancellation of most tests. We acknowledge that optimal validation of this algorithm would require randomly selecting and testing rejected specimens during a phase of high disease prevalence. Although low disease prevalence during our study period precluded validation testing, we recommend that such testing be performed.

Our systematic approach to screening requests to test for agents with the potential to threaten global health can prevent arbitrary decision making, reduce inappropriate testing, and increase the value of laboratory consultation. The principles guiding our testing protocols for SARS and avian influenza can be generalized to future global health threats. Responsible and judicious use of diagnostic testing will be crucial for minimizing the risk of providing clinicians with misleading results that could severely disrupt the public health system and lead to an unnecessary expenditure of limited resources. With the emergence of highly pathogenic avian influenza, we anticipate further demands on laboratory and public health resources that will necessitate effective, pragmatic tools to enhance the value of laboratorian-clinician consultation before tests are performed on site or referred to an LRN laboratory.
